# Emerging Developments in Management of Melanoma During the COVID-19 Era

**DOI:** 10.3389/fmed.2021.769368

**Published:** 2021-11-08

**Authors:** Andraia R. Li, Manuel Valdebran, Daniel Y. Reuben

**Affiliations:** ^1^Department of Dermatology and Dermatologic Surgery, Medical University of South Carolina, Charleston, SC, United States; ^2^Department of Pediatrics, Medical University of South Carolina, Charleston, SC, United States; ^3^Department of Medicine, Medical University of South Carolina, Charleston, SC, United States

**Keywords:** melanoma, coronavirus, SARS-CoV-2, malignant melanoma, COVID-19

## Abstract

In March 2020, the designation of the COVID-19 outbreak as a worldwide pandemic marked the beginning of an unprecedented era in modern medicine. Facing the possibility of resource precincts and healthcare rationing, leading dermatological and cancer societies acted expeditiously to adapt their guidelines to these contingencies. Melanoma is a lethal and aggressive skin cancer necessitating a multidisciplinary approach to management and is associated with significant healthcare and economic cost in later stages of disease. In revisiting how the pandemic transformed guidelines from diagnosis and surveillance to surgical and systemic management of melanoma, we appraise the evidence behind these decisions and their enduring implications.

## Introduction

Cutaneous melanoma is the fifth most commonly diagnosed malignancy in the United States, and the most lethal cutaneous cancer (1, 2). The treatment of advanced and metastatic melanoma requires a multidisciplinary team of specialists and multimodal regimens, with later stages of disease associated with significant healthcare and economic burden (1, 3). Emergence of the COVID-19 pandemic broached an unprecedented need for judicious rationalization and allocation of healthcare resources worldwide (4). In response, governing bodies released new guidelines on the management of melanoma in the COVID-19 era, shaped with a greater consciousness for minimizing patient exposure to infection and reducing healthcare consumption in mind. While in some geographical areas this has abated and vaccination rates are improving, new variants pose a risk to patients and healthcare delivery methods should variants evade the effectiveness of current vaccines. Here, we review these new guidelines, the evidence behind them and the potential implications of these recommendations as well as possible remedies.

## Developments in Screening, Diagnosis, and Disease Surveillance

### Screening

Early detection of melanoma is imperative for survival but restrictions to outpatient services from March to June 2020 in response to the SARS-CoV-2 pandemic resulted in a significant drop in skin cancer screenings (5). With the cessation of screenings, questions have been raised about resuming these preventative practices in the post-COVID-19 era. To date modifications of screening recommendations during the COVID pandemic have stemmed from theoretical concerns not directly from data on viral exposure or outcome data (5). Thus, it is not clear that a change in current practice is yet warranted so long as safe patient care can be provided. The American Academy of Dermatology (AAD), the leading representative dermatological society in the United States, continues to advocate for routine screenings in their guidelines and their SPOT ME Skin Cancer campaign, with recommendations for in-person screenings in compliance with local and state Center for Disease Control and Prevention (CDC) guidelines (6). Additionally, the AAD, jointly with the Skin Cancer Foundation, endorsed continuation of self-skin examinations and application of the ABCDEs of melanoma (6, 7).

### Diagnosis

Given that the diagnosis of melanoma is primarily made on skin exams, delays in screening have raised concerns for ensuing delays in diagnosis (8). The long-term consequences of the COVID-19 pandemic on survival outcomes in melanoma are effectively unknown. A study conducted by the University of Pennsylvania Dermatopathology Department found no overall difference in median Breslow thickness or T staging at time of diagnosis between the pre-COVID-19 and COVID-19 era cohorts (9). However, surgical candidates had higher median thickness and higher proportions of T3 and T4 lesions at time of diagnosis than patients from the pre-COVID era (9).

Moreover, the pandemic prompted a substantial increase in the use of telemedicine services. In a survey of International Dermoscopy Society members, there was a reported 83.3% increase in teleconsultations (10). Despite an increase in utilization of these services, 57% of total respondents recounted making zero diagnoses of melanoma, raising concerns for an increase in missed cases during this time (10). On March 6, 2020, The National Comprehensive Cancer Center (NCCN) recommended that all new patients be evaluated with telehealth when possible, with a subsequent complete history and physical on the day of surgery if necessary (11). The goal, it would seem, was to reduce in-person exposure risks. A trade-off is if modification to a treatment plan is required when the patient arrives. Additionally, if telehealth is determined to be inferior for this purpose, as suggested by data, a future increase in delayed diagnoses or upstaged melanoma may occur (11). Aside from screening, diagnostic evaluations of an obvious, perhaps self-reported, lesion could be inaccurate through telemedicine. Further data collection to assess the accuracy of telemedicine compared with in-person diagnostic evaluation would be helpful in order to interpret recommendations for or against telehealth in this setting.

A potential solution for improving diagnostic accuracy is through the integration of imaging techniques with telemedicine services. Total body photography (TBP) is a commonly used non-invasive imaging technique for the photographic assisted detection of melanoma (12). Data has shown integration of TBP and dermoscopy with telemedicine services ensues a number-needed-to-biopsy (NNB) per one case of melanoma comparable to previously published reports for in-person encounters with dermatologists and physician assistants (13, 14). Additionally, prospective results found inclusion of TBP and sequential digital dermoscopy imaging to surveillance protocols aided clinicians in detecting the majority of new lesions in high-risk patients (15). Moreover, these outcomes are likely to be improved with the integration of artificial intelligence. Despite being in its nascent stages of development, diagnostic efficacy through machine learning have been comparable to that of trained clinicians, indicating these technical advances hold significant promise in enhancing the efficacy of image-based diagnostics (16).

### Surveillance

For patients with a history of melanoma, clinical surveillance can be delayed for 3–6 months in patients with asymptomatic localized disease (e.g., stages 0–II) or asymptomatic resected stage III disease, in the absence of concurrent systemic therapies, according to modified NCCN guidelines (11). In the setting of asymptomatic stage IIB/IIC melanoma, follow-up imaging can be deferred for 3–6 months (11). As screening guidelines have given wide latitude regarding frequency these modifications for surveillance screening are reasonable.

Additionally, the NCCN's adjusted guidelines related to patients on active therapy as well. Here, in the setting of adjuvant therapy, restaging was suggested to be delayed for upwards of 3 months (11). A clinician actively treating such patients need to use judgement regarding this proposed modification. The previous intention of restaging amidst adjuvant treatment was to ensure that the therapy is effective. Delaying that evaluation only continues to place the patient at risks and side-effects of the therapy without knowledge of its benefit. Since intravenous immunotherapy still obligates the patient to be available in-person repetitively every few weeks delaying restaging only reduces exposure to the Radiology department—a small imperceptible change in risk status but potentially with larger consequences should disease progression occur undetected.

## Developments in Surgical Management

### Local Wide Excision and Sentinel Lymph Node Biopsy

Consensus to delay LWE for up to 3 months for new cases of melanoma *in situ* and stage T1 melanoma was ubiquitous across various associations, including the NCCN, American College of Mohs Surgery, British Association of Dermatologist (BAD) and British Society for Dermatological Surgery (BSDS) (11, 17, 18). The NCCN endorsed deferring LWE for up to 3 months in patients with T1 melanoma, even in the setting of positive margins, in the absence of observable residual disease (11). However, larger enduring lesions should be excised in an office setting (11).

Current evidence on the association between surgical timing from excisional biopsy to LWE and survival have been inconsistent (18). A retrospective study of patients with cutaneous melanoma found time from excisional biopsy to LWE did not result in meaningful differences in overall survival (OS) and disease free survival (DFS) between surgical groups (19). However, analyses of patients with stage I–III melanoma in the National Cancer Database (NCBD) found LWE within 60 days of diagnosis granted a modest survival advantage while LWE ≥90 days after initial biopsy was associated with increased mortality (20, 21). Additional prospective studies are needed to ascertain the effect of surgical timing on survival outcomes given the limitations of retrospective studies.

On March 24, 2020, the European Society for Medical Oncology (ESMO) published their own guidelines on the management of melanoma in the COVID-19 era, stratifying patients into high, high to medium, and low priority treatment groups (22). LWE and sentinel LN biopsy were recommended for all patients with invasive T1b disease or higher, with T3 and T4 lesions assigned high priority and T1 and T2 lesions designated medium priority for excision (22). In the U.S., the NCCN recommended discussing sentinel LN biopsy for lesions of stage T1b or higher, with the potential for delaying LN biopsies for up to 3 months unless LWE in an operating setting is planned (11). These recommendations were formulated to reduce patient and staff exposures. Also early in the pandemic a shortage of supplies and resources was either real or perceived. As understanding of infection risks, mitigation thereof, improved delivery of supplies and vaccinations programs have been carried out and resumption of surgical services have occurred. Surgical guideline modifications may not need to be as stringent moving forward. Furthermore, delay of definitive surgery can lend to increased patient anxiety and would require careful patient counseling in this situation.

### Resections and Lymphadenectomies

The NCCN advocated for deferring therapeutic lymphadenectomies for palpable LN, and offering neoadjuvant therapy, including immune-checkpoint inhibitors (ICIs) or BRAF/MEK inhibitors, instead (11). However, in the absence of available adjuvant therapies, the British Association of Plastic and Reconstructive Aesthetic Surgeons (BAPRAS) considered lymphadenectomies a viable primary treatment for achieving local control for recurrent nodal disease (23). For non-metastatic stage III melanoma, surgical resection should be performed 8–9 weeks following initiation of neoadjuvant therapy according to modified NCCN guidelines (11). Additionally, resections of metastatic stage III and IV disease should be deferred, unless the patient is critical or symptomatic, with continuation of systemic monotherapy instead (11). ESMO considered curative resections of stage III lesions, surgery for patients on neoadjuvant therapies, and management of surgical complications as high priority, but recognized delaying surgery is acceptable as it has not been shown to influence survival in many cases (22).

### Radiotherapy

Patients with stage IV disease and brain metastases are high-priority for radiotherapy according to ESMO guidelines (22). In accordance, the NCCN guidelines recommended stereotactic radiosurgery as initial treatment for patients with symptomatic or steroid-dependent metastatic disease and endorses discontinuation of, or tapering steroids when initiating ICIs (11). Evidence for ICIs in patients with metastatic melanoma after stereotactic radiosurgery (SRS) have been reported in several retrospective studies but these findings have been inconsistent and additional prospective studies are still ongoing (24–27). With respect to radiotherapy of brain metastases amidst the pandemic, it is difficult to advise modification of this treatment modality as there is not an equivalent for it. Diligent screening of patient symptoms, rapid COVID testing and use of PPE is imperative in this case.

## Developments in Systemic Treatments

### Neoadjuvant Therapies—Immune Checkpoint Inhibitors

Consideration for the possibility of resource limitations was commonly addressed across multiple guidelines, especially in the case of neoadjuvant therapies. Although the NCCN recognized that neoadjuvant therapy is not superior to combination surgery and adjuvant therapy, neoadjuvant therapy for primary management of stage III disease may be a judicious option in the setting of resource limitations (11). For neoadjuvant ICI, the NCCN and ESMO both recommended a regimen of higher dose pembrolizumab at 400 mg every 6 weeks or nivolumab at 480 mg every 4 weeks (11, 22). On April 28, 2020, the FDA approved the accelerated regimen of pembrolizumab following the results of the KEYNOTE-55 trial (28). Interim analysis found 400 mg of pembrolizumab every 6 weeks was comparable to the original regimen of 200 mg every 3 weeks (29). An accelerated regimen is advantageous as longer intervals between cycles minimizes exposures.

Regarding dual therapy, the NCCN, ESMO and BAPRAS recommended clinicians exercise caution when starting combination ICI regimens (11, 22, 23). Results of Checkmate-067 found combination nivolumab-ipilimumab therapy significantly prolonged OS than nivolumab or ipilimumab alone (60.0 vs. 36.9 vs. 19.9 months) but correspondingly produced increased rates of grade ≥3 adverse events (AE) from 20–30% to 50–60% (30).

Immune-related AE (irAE) are due to an augmented immune response secondary to ICI therapy (31). Immunosuppressants are frequently used to temporarily attenuate the immune response, but can promote an increased risk for COVID-19 infections (8, 32). Pneumonitis can be a confounding toxicity that can mimic an active SARS-CoV-2 infection with symptoms such as shortness of breath, cough and dyspnea (33). The NCCN recommended COVID-19 testing if a diagnosis of pneumonitis was suspected prior to initiation of steroids (11). While this was a reasonable recommendation, in practice patients have not always been able to expeditiously schedule testing or receive quick results depending on their locale. A potential delay in treatment of pneumonitis can have high morbidity and exemplifies an unintended negative outcome that new recommendations can promote. For routine monitoring of patients on ICIs, the ESMO, and BAPRAS both endorsed routine telemedicine visits, and labs at healthcare facilities equipped with appropriate COVID-19 precautions (22, 23).

Taking into consideration the risk and benefits, the decision for initiation of dual ICI therapy should be made on an individual basis according to the NCCN and ESMO (11, 22). The BAPRAS recommended monotherapy in the setting of metastatic disease for all but high risk patients (23). Likewise, the NCCN endorsed dual ICI therapy for stage IV disease with brain metastases, citing superior intracranial tumor response to ICIs (11). A number of phase II trials have shown improved response rates of brain metastases associated with dual immunotherapy over other agents but the phase III NIBIT-M2 trial assessing ICIs in the treatment of melanoma brain metastases is still ongoing (34, 35).

For stage IV disease, a regimen consisting of nivolumab 1 mg/kg and ipilimumab 3 mg/kg (NIVO1+IPI3) for four cycles has been established (11). An alternative regimen of nivolumab 3 mg/kg and ipilimumab 1 mg/kg (NIVO3+IPI1) may be considered if there is notable concern for irAE according to the ESMO and NCCN (11, 22). These recommendations were based on the results of CheckMate 511, which showed the alternative regimen of NIVO3+IPI1 decreased the incidence of grade 3–5 AEs (34 vs. 48%), with no meaningful difference between median progression free survival (PFS) (9.9 vs. 8.9 months) or overall response rate (45.6 vs. 50.6%) compared to the prior NIVO1+IPI3 regimen (32, 36). Applying the alternate dosing strategy will substantially reduce dual ICI risks during the pandemic.

In the setting of a SARS-CoV-2 infection, patients can resume immunotherapy once fully recovered or after 10 days from last presentation of symptoms under the BAD and BSDS guidelines (17). There is currently no clear evidence that use of ICIs worsens outcomes of COVID-19 infections (37–39). Nonetheless, there is evidence to suggest ICIs may be discontinued in patients with metastatic melanoma who achieved complete remission with PD-1 blockade (40). Follow up analysis of KEYNOTE-001 showed patients with melanoma who discontinued pembrolizumab after complete response to PD-1 blockade had comparable rates of DFS to that of all complete responders (e.g., including those who continued ICI therapy) at 24 months (89.9 vs. 90.9%) (40).

### Neoadjuvant Therapies—Targeted Therapy

The ESMO considered targeted therapy high priority in patients with non-operable stage III and IV disease (22). The NCCN recommended a regimen of BRAF/MEK inhibitors for 8 weeks followed by surgery in the setting of neoadjuvant therapy (11). Specifically, the BAPRAS recommended combination encorafenib and binimetinib, given these agents are less likely to mimic the symptoms of SARS-CoV-2 infections compared to ICIs (23). The most common grade 3–4 AEs associated with dual BRAF/MEK inhibitor therapy include elevated gamma-glutamyl transferase (9%), creatine phosphokinase (7%), and hypertension (6%) (41).

### Adjuvant Therapies

Adjuvant therapy can be delayed for up to 12 weeks in accordance with NCCN and ESMO guidelines (11, 22). This seemed reasonable given that trial design which established adjuvant therapy allowed for this type of delay in most cases (42, 43). Patients with high-risk stage III disease, defined as sentinel LN deposit >1 mm or stage >IIIa disease, are considered high to medium priority for adjuvant therapy according to ESMO (22). In contrast, the BAPRAS only recommended adjuvant therapy in the setting of stage IIIc, IIId, and IV disease, but not in stage IIIa or IIIb cases (23). Restricting adjuvant therapy by these guidelines appears arbitrary and undoubtedly will lead to a reversal in average OS gains. It also contradicts the aforementioned consideration of starting neoadjuvant therapy on advanced stage melanoma patients in order to briefly postpone surgery.

Depending on hospital operations and resources, ESMO advised physicians to consider starting patients on a BRAF/MEK inhibitor given the ease of oral dosing, with a potential for transition to intravenously routed immunotherapies later on (22). Currently, there are no head to head trials comparing survival outcomes of adjuvant BRAF/MEK inhibitors with adjuvant ICIs for resected stage III melanomas (44). In the COMBI-AD trial, patients treated with combination BRAF/MEK inhibitor therapy of dabrafenib plus trametinib had an estimated 58% relapse-free survival rate at 3 years, compared to 39% with placebo (45). However, high rates of fever (63%) and chills (37%), as well as other flu-like symptoms associated with dabrafenib plus trametinib may make this combination counterintuitive (45). Such symptoms amidst a viral pandemic could be confounding, leading to anxiety and increased in-person resource use. Comparably, KEYNOTE-054 showed patients with resected stage III melanoma treated with adjuvant pembrolizumab had a 64% relapse-free survival rate, compared to 44% in placebo group at 3-year median follow up (42). Given that immunotherapy is not likely to cause fevers and chills, it is especially attractive at the present tine as an adjuvant strategy having similar efficacy on cross trial comparison to combination BRAF/MEK inhibitor therapy.

## Conclusions

The COVID-19 pandemic swept in a period of uncertainty and forced clinicians to rethink the existing paradigms in treatment of melanoma to minimize both healthcare consumption and exposure. The unforeseen nature of the pandemic required societies to act quickly and swiftly to enact provisional guidelines and served as a catalyst for adapting new applications such as telemedicine into routine practice. A general impetus has been to limit patient exposure, reduce durable supply use and allow for redeployment of medical resources in a priority manner. How this will affect patient care will be the subject of review for years to come. The data generated during the pandemic to date is likely not robust enough to merit recommending long-term practice changes. Yet, despite the provisional nature of these guidelines, the COVID-19 pandemic highlighted many opportunities for optimization in our healthcare system. For instance telehealth may become more wide-spread and potentially could include software technology to assist in improving diagnostic accuracy. Neoadjuvant therapy, if appropriate, can defer surgery and operating room risks and resources, potentially until a pandemic has subsided or resources and PPI restocked. Systemic therapies have been scrutinized and compared to assess efficacy and side-effects. Clinical judgement on selecting these and the appropriate dose and schedule is still important. Despite best efforts some recommendations can be controversial in producing unintended consequences. As shown one recommending body may conflict with another. Hopefully ongoing efforts will provide input on cancer patient outcomes on and off therapy during this pandemic (46). Until further evidence based data is available clinicians will be challenged to modify cancer care safely for their patients' welfare.

## Author Contributions

AL: provided the conception and was a major contributor in writing the manuscript. MV: drafted and revised the manuscript critically for important intellectual content. DR: was a major contributor in writing the manuscript and revised it critically for intellectual content. AL, MV, and DR: provided final approval of the version of the manuscript to be published and agree to account for all aspects of work in ensuring accuracy and integrity. All authors contributed to the article and approved the submitted version.

## Conflict of Interest

The authors declare that the research was conducted in the absence of any commercial or financial relationships that could be construed as a potential conflict of interest.

## Publisher's Note

All claims expressed in this article are solely those of the authors and do not necessarily represent those of their affiliated organizations, or those of the publisher, the editors and the reviewers. Any product that may be evaluated in this article, or claim that may be made by its manufacturer, is not guaranteed or endorsed by the publisher.
